# α_v_β_6_-Targeted Molecular PET/CT Imaging of the Lungs After SARS-CoV-2 Infection

**DOI:** 10.2967/jnumed.120.255364

**Published:** 2020-12

**Authors:** Cameron C. Foster, Ryan A. Davis, Sven H. Hausner, Julie L. Sutcliffe

**Affiliations:** 1 Division of Nuclear Medicine, Department of Radiology, University of California Davis, Sacramento, California; 2 Division of Hematology/Oncology, Department of Internal Medicine, University of California Davis, Sacramento, California; and; 3 Department of Biomedical Engineering, University of California Davis, Davis, California

**Keywords:** integrins, SARS-CoV-2, positron emission tomography, peptides, fibrosis

## Abstract

The true impact and long-term damage to organs such as the lungs after severe acute respiratory syndrome coronavirus 2 (SARS-CoV-2) infection remain to be determined. Noninvasive molecularly targeted imaging may play a critical role in aiding visualization and understanding of the systemic damage. We have identified α_v_β_6_ as a molecular target; an epithelium-specific cell surface receptor that is low or undetectable in healthy adult epithelium but upregulated in select injured tissues, including fibrotic lung. Herein we report the first human PET/CT images using the integrin α_v_β_6_-binding peptide (^18^F-α_v_β_6_-BP) in a patient 2 mo after the acute phase of infection. Minimal uptake of ^18^F-α_v_β_6_-BP was noted in normal lung parenchyma, with uptake being elevated in areas corresponding to opacities on CT. This case suggests that ^18^F-α_v_β_6_-BP PET/CT is a promising noninvasive approach to identify the presence and potentially monitor the persistence and progression of lung damage.

As coronavirus disease 2019 (COVID-19) continues to spread through the global population, the burden of diseases such as fibrotic lung after acute COVID-19 is unknown, and close follow up of patients is critical (1). The integrin subtype α_v_β_6_ is an epithelium-specific receptor found at low and generally undetectable levels in healthy adult epithelium, is known to be upregulated during tissue remodeling, is a potent activator of transforming growth factor β-1, and plays a key role in the progression of numerous fibrotic diseases, including lung, liver, and kidney fibrosis (2–5). We and others have developed radiolabeled peptides to noninvasively image integrin α_v_β_6_ expression (6–8). A recent study by Lukey et al. evaluated ^18^F-FB-A20FMDV2 PET in healthy and fibrotic lung and concluded that lung uptake of ^18^F-FB-A20FMDV2 was markedly increased in subjects with pulmonary fibrosis as compared with healthy volunteers (8). In our study using ^18^F-α_v_β_6_–binding peptide (^18^F-α_v_β_6_-BP) PET/CT (NCT03164486) in patients with cancer, immunohistochemical analysis of biopsy specimens confirmed high expression of integrin α_v_β_6_ for tissues showing high uptake on ^18^F-α_v_β_6_-BP PET (7). Taken together, given the role of integrin α_v_β_6_ as a potent activator of transforming growth factor β-1 and its role in the progression of numerous fibrotic diseases, including those of the lung, liver, and kidney, we propose to extend the use of the demonstrated integrin α_v_β_6_ imaging agent ^18^F-α_v_β_6_-BP to assess lung damage in patients after severe acute respiratory syndrome coronavirus 2 (SARS-CoV-2) infection. Here, we present the first molecularly targeted ^18^F-α_v_β_6_-BP PET images obtained in a patient 2 mo after SARS**-**CoV**-**2 infection and correlate them with CT images.

## MATERIALS AND METHODS

This study protocol was approved by the University of California Davis Institutional Review Board (FWA00004557), and prior written informed consent was obtained from the patient. This is the first case report of a patient included in the ^18^F-α_v_β_6_-BP PET/CT COVID-19 imaging trial (ClinicalTrials.gov NCT04376593). The study was conducted following U.S. Common Rule. The primary objective of this study was to determine the safety and feasibility of ^18^F-α_v_β_6_-BP PET to detect the presence and monitor the regression or progression of lung damage in patients after SARS-CoV-2 infection. Up to 10 patients with a prior diagnosis of SARS-CoV-2 infection who have since tested negatively will be recruited to the study. Each participant will undergo up to 3 ^18^F-α_v_β_6_-BP PET/CT scans over a 6-mo time frame. The specific aims are to acquire ^18^F-α_v_β_6_-BP PET/CT images in patients diagnosed with SARS-CoV-2, to demonstrate ^18^F-α_v_β_6_-BP accumulation in lung damage, to establish that ^18^F-α_v_β_6_-BP accumulation correlates with the regression or progression of lung damage over time, and to correlate ^18^F-α_v_β_6_-BP accumulation in the lung to lung damage as indicated on CT. ^18^F-α_v_β_6_-BP was manufactured in compliance with current good manufacturing practices under the guidelines of U.S. Pharmacopeia chapter <823> as previously described (7).

### Subject History

**FIGURE 1.**
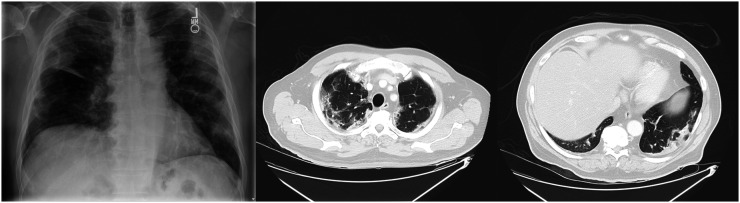
Initial chest radiograph at hospital admission showing diffuse pulmonary opacities in mid and peripheral lungs bilaterally (left), and transaxial CT scans of upper lung (middle) and lower lung (right) showing areas of ground-glass and consolidative changes on day 4 after admission.

A 71-y-old man with a prior history of hypertension developed respiratory symptoms, tested positively for SARS-CoV-2 (reverse-transcription polymerase chain reaction nasopharyngeal swab), and was subsequently admitted to the hospital. He was treated for hypoxia and superimposed bacterial pneumonia and received supplemental oxygen and antibiotics but was not intubated. The patient’s chest radiograph at admission to the hospital showed diffuse pulmonary opacities in the mid and peripheral lungs bilaterally (Fig. 1), consistent with a diagnosis of SARS**-**CoV**-**2–associated pneumonia. The chest CT scan of the thorax 4 d later showed moderate to severe bilateral central and peripheral patchy areas of ground-glass and consolidative changes throughout the lungs. After testing negatively twice for COVID-19 (approximately 2 mo after the initial positive test), the patient was enrolled in the ^18^F-α_v_β_6_-BP PET/CT COVID-19 imaging trial.

### Imaging

^18^F-α_v_β_6_-BP PET/CT images were acquired during recovery 66 d after the initial chest CT scan. The patient was injected with ^18^F-α_v_β_6_-BP (340 MBq) as a rapid intravenous bolus. Immediately before and after the injection, the patient’s vital signs (blood pressure, heart rate, pulse oximetry value, and body temperature) were measured. The patient rested for 1 h before the PET/CT scan. The PET scan was performed on a GE Discovery 690 PET/CT scanner at 2 min per bed position. A PET/CT acquisition of the thorax with arms up was performed with a typical low-dose CT scan (140 kV “smart mA” [50–350 mA]; noise index, 20) of the thorax for attenuation correction. Immediately afterward, a second non–attenuation-corrected PET scan was acquired from the skull vertex to the proximal thighs with arms up.

### Data Analysis

Reconstructed PET/CT images were displayed using Advantage Workstation Client (server 3.2 ext3; VolumeViewer 14 ext4; GE Healthcare) and reoriented into maximum-intensity-projection transaxial, coronal, and sagittal images. PET, fused PET/CT, and CT images were reviewed. The PET images were interpreted qualitatively and semiquantitatively. Semiquantitative analysis included regions of interest placed around tracer-avid foci suggestive of lung damage to obtain SUV_max_ and SUV_mean_.

## RESULTS

**FIGURE 2.**
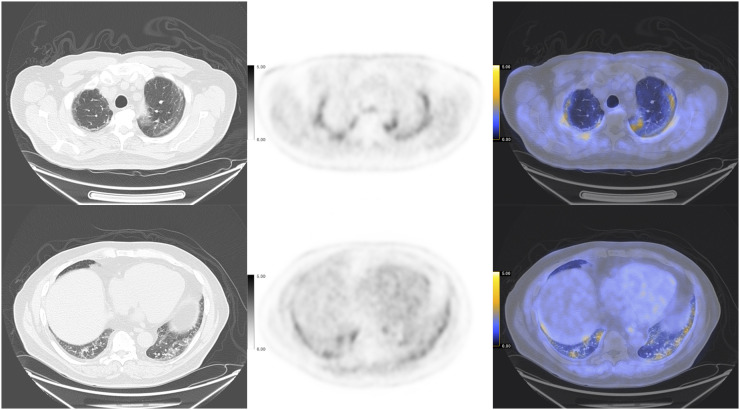
Transaxial CT (left), attenuation-corrected ^18^F-α_v_β_6_-BP PET (middle; scale: SUV_max_ of 5), and fused ^18^F-α_v_β_6_-BP PET/CT (right) images through upper lungs (top) and lower lungs (bottom), showing increased uptake and areas of bilateral patchy opacity.

No changes in vital signs were noted during the study, and the patient’s SpO_2_ was 100%. The transaxial CT images (Fig. 2) through both the upper and the lower lungs showed improved areas of bilateral patchy opacities as compared with the initial chest CT (Fig. 1). Transaxial coregistered attenuation-corrected ^18^F-α_v_β_6_-BP PET images through the upper and lower lungs (scale: SUV_max_ of 5.0; Fig. 2) demonstrated elevated uptake of ^18^F-α_v_β_6_-BP (SUV_max_ of 3.0) in areas corresponding to areas of opacity noted on CT. Concurrently, regions of normal lung parenchyma seen on CT demonstrated low levels of ^18^F-α_v_β_6_-BP uptake on PET, with an SUV_max_ of 0.8–1.0.

## DISCUSSION

The long-term systemic health impact of COVID-19 in both symptomatic and asymptomatic subjects is yet to be determined. In the future, it will be critically important to noninvasively evaluate the persistence and potential progression of abnormalities of the lung and other organs. As was recently described by George et al., long-term follow-up studies to establish the true prevalence of post–COVID-19 fibrosis are essential, and these preliminary data suggest that ^18^F-α_v_β_6_-BP PET/CT is a promising noninvasive strategy to address this issue (1).

Although anatomic imaging with CT of patients infected with SARS**-**CoV**-**2 often shows a mix of consolidation and ground-glass opacities in the lungs, early identification is often confounded by delayed radiographic presentations (9). In addition, lung damage may be missed in the large fraction of asymptomatic patients. Long et al. recently reported the clinical and immunologic assessment of asymptomatic patients; in radiologic and laboratory findings, they noted that 11 of 37 patients showed focal ground-glass opacities on CT and that 14 of 21 patients had abnormal radiologic findings in at least one lung (10). Several incidental findings of COVID-19 have also been noted in patients undergoing ^18^F-FDG PET/CT studies for routine oncologic indications (11).

Considering that the anatomic observations made by CT are caused by major changes in the tissue, molecularly targeted noninvasive imaging strategies, such as ^18^F-α_v_β_6_-BP PET, can provide essential complementary clinical information to further understand the nature of the underlying tissue remodeling resulting in these changes. The quantitative information from the ^18^F-α_v_β_6_-BP PET scans could help identify potential damage sooner (before clinical or symptom manifestation) and ascertain if the damage is transient or progressive. Molecular imaging can thus also contribute to improved clinical detection and the longitudinal study of recovery from damage to lungs as well as other organs after infection. Other radiopharmaceuticals, such as ^18^F-FDG and ^18^F-fibroblast activation protein inhibitor (12), could also provide complementary information about SARS**-**CoV**-**2 infection.

The integrin α_v_β_6_ has previously been described as a potential biomarker of fibrotic lung diseases, including idiopathic pulmonary fibrosis, nonspecific interstitial pneumonitis, and chronic hypersensitivity pneumonitis (6,8), and has been recognized as an important activator of transforming growth factor β-1 during tissue remodeling (2,3). Semiquantitative analysis of integrin α_v_β_6_ expression in lung biopsy specimens from individuals with idiopathic pulmonary fibrosis has been shown to have potential prognostic significance, with higher levels predicting more rapid progression and mortality (8).

We and others have developed radiolabeled peptides to image integrin α_v_β_6_ expression (6–8). A recent study by Lukey et al. evaluated ^18^F-FB-A20FMDV2 in healthy and fibrotic lung and concluded that lung uptake of ^18^F-FB-A20FMDV2 was markedly increased in subjects with pulmonary fibrosis as compared with healthy volunteers (SUV_mean_, 1.03 and 0.54, respectively) (8). In our study using ^18^F-α_v_β_6_-BP (NCT03164486) in patients with cancer, PET images showed significant uptake of ^18^F-α_v_β_6_-BP in both the primary lesion and metastases, including metastasis to brain, bone, liver, and lung; immunohistochemical analysis of biopsy specimens confirmed high expression of integrin α_v_β_6_ for tissues showing high uptake on ^18^F-α_v_β_6_-BP PET (7). SUV_max_ in the primary tumors and metastases was as high as 25.0, whereas low levels of uptake were noted in normal lung parenchyma (SUV_max_ ≤ 1.0; range, 0.3–1.0).

This preliminary study has shown a correlation of integrin α_v_β_6_–targeted ^18^F-α_v_β_6_-BP PET with lung damage identified by CT. For areas of lung that corresponded to SARS**-**CoV**-**2–related ground-glass opacities and consolidation by CT, the SUV_max_ observed by ^18^F-α_v_β_6_-BP PET was approximately 3.0. These values are 3 times those reported by Lukey et al. (8) for ^18^F-FB-A20FMDV2 in fibrotic lung and represent an almost 4-fold increase in uptake of ^18^F-α_v_β_6_-BP in abnormal versus normal lung tissue and clear visualization of damage. These observations suggest that ^18^F-α_v_β_6_-BP PET/CT is a promising strategy to detect and monitor the development and progression of lung fibrosis after SARS**-**CoV**-**2 infection and to further understand the nature of the tissue remodeling and progression in recovering patients over time. The main limitation of this study is the single imaging time point; follow-up ^18^F-α_v_β_6_-BP PET/CT scans will be critically important to evaluate the persistence and potential progression of abnormalities of the lung and other organs. ^18^F-α_v_β_6_-BP PET/CT scans are currently scheduled for 3 and 6 mo, and a total of 10 patients will be enrolled in the study. These longitudinal studies are needed to determine the ability of this imaging test to predict and monitor post–COVID-19 lung fibrosis.

## CONCLUSION

As COVID-19 continues to spread through the global population, the burden of diseases such as fibrotic lung after acute COVID-19 is yet to be determined and close follow-up of patients is critical. This study has shown a correlation between integrin α_v_β_6_–targeted ^18^F-α_v_β_6_-BP PET and lung damage identified by CT; we therefore will further investigate the role of ^18^F-α_v_β_6_-BP as a PET imaging agent for early detection of lung damage and monitoring of disease progression.

## DISCLOSURE

This work was funded in part through NIH U01 CA217665. Julie Sutcliffe and Sven Hausner are named inventors on WO2015160770. Julie Sutcliffe is the founder and a stock holder of Luminance Biosciences, Inc. Luminance Biosciences, Inc., has licensed WO2015160770. No other potential conflict of interest relevant to this article was reported.

**KEY POINTS**

**QUESTION:** Can ^18^F-α_v_β_6_-BP PET detect damage to the lungs after SARS**-**CoV**-**2 infection?
**PERTINENT FINDINGS:** ^18^F-α_v_β_6_-BP PET is a noninvasive approach to identify the presence and potentially monitor the persistence and progression of lung damage.
**IMPLICATIONS FOR PATIENT CARE:** This approach has the potential not only to detect organ damage but also to guide and monitor response to novel molecularly targeted treatments.
